# Insect Herbivory Caused Plant Stress Emissions Increases the Negative Radiative Forcing of Aerosols

**DOI:** 10.1029/2022JD036733

**Published:** 2022-07-12

**Authors:** E. Holopainen, H. Kokkola, C. Faiola, A. Laakso, T. Kühn

**Affiliations:** ^1^ Atmospheric Research Centre of Eastern Finland Finnish Meteorological Institute Kuopio Finland; ^2^ Aerosol Physics Research Group University of Eastern Finland Kuopio Finland; ^3^ Department of Ecology and Evolutionary Biology University of California Irvine Irvine CA USA; ^4^ Department of Chemistry University of California Irvine Irvine CA USA

**Keywords:** global modeling, secondary organic aerosol, volatile organic compound, plant stress, radiative forcing, aerosol‐cloud interactions

## Abstract

Plant stress in a changing climate is predicted to increase plant volatile organic compound (VOC) emissions and thus can affect the formed secondary organic aerosol (SOA) concentrations, which in turn affect the radiative properties of clouds and aerosol. However, global aerosol‐climate models do not usually consider plant stress induced VOCs in their emission schemes. In this study, we modified the monoterpene emission factors in biogenic emission model to simulate biotic stress caused by insect herbivory on needleleaf evergreen boreal and broadleaf deciduous boreal trees and studied the consequent effects on SOA formation, aerosol‐cloud interactions as well as direct radiative effects of formed SOA. Simulations were done altering the fraction of stressed and healthy trees in the latest version of ECHAM‐HAMMOZ (ECHAM6.3‐HAM2.3‐MOZ1.0) global aerosol‐climate model. Our simulations showed that increasing the extent of stress to the aforementioned tree types, substantially increased the SOA burden especially over the areas where these trees are located. This indicates that increased VOC emissions due to increasing stress enhance the SOA formation via oxidation of VOCs to low VOCs. In addition, cloud droplet number concentration at the cloud top increased with increasing extent of biotic stress. This indicates that as SOA formation increases, it further enhances the number of particles acting as cloud condensation nuclei. The increase in SOA formation also decreased both all‐sky and clear‐sky radiative forcing. This was due to a shift in particle size distributions that enhanced aerosol reflecting and scattering of incoming solar radiation.

## Key Points

- Increasing plant stress enhanced secondary organic aerosol (SOA) formation due to increase in volatile organic compound emissions
- Enhanced biotic stress increased the number of particles acting as cloud condensation nuclei and thus cloud droplet number concentration
- Increased SOA formation enhanced aerosol scattering which decreased all‐sky and clear‐sky radiative forcing

## 1 Introduction

Atmospheric aerosols affect the climate by scattering and absorbing solar radiation directly (Seinfeld & Pandis, 2006). In addition, they have a possibility to go through aerosol‐cloud‐interactions impacting the lifetime, formation, and the radiative properties of the clouds (Albrecht, 1989; Kerminen et al., 2005; Makkonen et al., 2009; Twomey, 1991). As the magnitude of the climate effects of atmospheric aerosol particles are uncertain, it causes a large uncertainty to the estimation of radiation budget of the Earth (IPCC, 2021).

One source of uncertainty in estimating the climate effects of anthropogenic aerosol is that the sensitivity of radiative forcing (RF) to perturbations by anthropogenic aerosol depends on the amount of natural background aerosol (Carslaw et al., 2013). One significant source of natural aerosol is vegetation as aerosol particles can be formed through oxidation of biosphere emitted volatile organic compounds (VOCs) (Donahue et al., 2013; Kulmala, Petäjä, et al., 2014; Schobesberger et al., 2013). VOCs are carbon‐structured molecules which evaporate in ambient conditions (Faiola & Taipale, 2020). A majority of the emitted VOCs originate from terrestrial vegetation such as trees and plants (Guenther et al., 2012; Jimenez et al., 2009; Sporre et al., 2020). These biogenic VOCs are highly reactive and play an important role in atmospheric chemistry processes (Carter, 1994; de Gouw et al., 2018; Griffith et al., 2016).

VOCs serve important ecological functions in plant defense and communication. Trees and plants emit VOCs to protect them from high temperature stress (Materić et al., 2015; Sharkey & Yeh, 2001) and to interact (such as attracting pollinators) with other organisms (Baldwin et al., 2002; Proffit et al., 2020). It has also been suggested that trees communicate with each other through emitting VOCs (Runyon et al., 2006; Ueda et al., 2012; Zebelo et al., 2012). VOC emissions can be altered by abiotic (non‐living) or biotic (living) plant stressors. Major abiotic plant stressors include extreme heat or drought (He et al., 2018). Major biotic plant stressors include insect herbivory or pathogens (J. K. Holopainen & Gershenzon, 2010; Zhao et al., 2017). Abiotic and biotic stress factors cause deviation from plants optimal living conditions and thus are known to alter the rate and spectrum of VOC emissions (Faiola & Taipale, 2020; J. K. Holopainen, 2004; J. K. Holopainen & Gershenzon, 2010; J. K. Holopainen et al., 2018; Niinemets, 2010). Especially the biotic stress factors can enhance the VOC emission rates of for example, monoterpene or sesquiterpene (Faiola & Taipale, 2020; Taipale et al., 2021).

Non‐refractory particles less than 1 micron in diameter are composed of 20%–90% organic aerosol (OA) with a high level of spatial and temporal variation (Jimenez et al., 2009; Murphy et al., 2006; Zhang et al., 2007). From these OA particles around 60%–70% is secondary organic aerosol (SOA) in a global scale (Goldstein & Galbally, 2007; Hallquist et al., 2009; Zhang et al., 2007). SOA is formed through oxidation of volatile, and semivolatile, organic species and the subsequent gas‐to‐particle partitioning of the oxidation products (Faiola & Taipale, 2020; Hallquist et al., 2009; Jimenez et al., 2009; Zhang et al., 2007). Consequently, increased plant stress VOC emissions can lead to enhanced formation of SOA with subsequent impacts on cloud formation and radiative transfer (Kerminen & Kulmala, 2002; Taipale et al., 2021). The sources, sinks, and atmospheric processing of SOA are uncertain which causes also uncertainty to the estimation of RF of aerosols in global climate models (Sporre et al., 2020; Tsigaridis et al., 2014). In addition, the contribution of biotic stress to VOC emissions and further to SOA formation is not usually considered in global aerosol‐climate models and thus more investigation is needed to more precisely evaluate the effects of SOA to atmosphere (Faiola & Taipale, 2020). However, there has been studies which state that biotic plant stress can massively influence the size and amount of formed SOA through enhancing of VOC emissions as well as affect the cloud condensation nuclei (CCN) concentrations in global model simulations. For example, Joutsensaari et al. (2015) found that increasing monoterpene emissions by 10‐fold, and assuming that 10% of the boreal forest area is experiencing biotic stress, increases the total particulate mass by 480% and CCN concentrations by 45% locally. In addition, Taipale et al. (2021) showed that biotic stress in plants is capable to perturb the amount and size of aerosol particles as well as increase the amount of newly formed particles. Unfortunately, these studies are using assumptions of biotic stress factors or limited to process level only and does not take into account the actual feedbacks of biotic stress induced SOA to radiative effects.

Here, we used the ECHAM‐HAMMOZ global aerosol‐climate model (Schultz et al., 2018) with sectional aerosol representation, SALSA (Kokkola, Kuhn, et al., 2018), and MEGAN v2.1 emission model (Guenther et al., 2012) to simulate the effects of biotic stress, due to insect herbivore infestation, on needleleaf evergreen boreal and broadleaf deciduous boreal trees in terms of SOA formation, clouds and radiative effects. The structure of the study is as follows. In Section 2 we present details of the global aerosol‐climate model, ECHAM‐HAMMOZ with SALSA microphysics package, and MEGAN v2.1 emission model used in this study. In the same section, we also present the values used for monoterpene emission factors and the details of the simulations performed in this study. In addition, we present reasoning of the time period and areas of interest chosen for this study. In Section 3 we present comparisons of simulations ranging from 10% to 100% stressed tree emissions to a baseline scenario where all of the trees were healthy. The comparison is done in terms of SOA burden, CDNC at cloud top and top of the atmosphere (TOA) clear‐sky and all‐sky RF in 2D map figures from 40° latitude northward. Finally we present field mean values from land area SOA burden, CDNC at cloud top and TOA clear‐sky and all‐sky RF as a function of increasing stress percentage in the simulations.

## 2 Materials and Methods

In this section we will describe the ECHAM‐HAMMOZ global aerosol‐climate model and the MEGAN v2.1 emission module, which were used in this study to simulate the effects of increasing VOC emissions, due to biotic stress in needleleaf evergreen boreal and broadleaf deciduous boreal trees, on SOA formation, clouds and radiative effects on a global scale. In addition, we will describe the different simulations, which were done using the ECHAM‐HAMMOZ global model, by altering the stress percentage of the trees.

### 2.1 ECHAM‐HAMMOZ

In this study we used the latest stable version of ECHAM‐HAMMOZ (ECHAM6.3‐HAM2.3‐MOZ1.0) (Schultz et al., 2018), which is a 3‐dimensional aerosol‐chemistry‐climate model. It consists of the host atmospheric model ECHAM (Stevens et al., 2013), aerosol model HAM (Kokkola, Kuhn, et al., 2018; Tegen et al., 2019), and chemistry model MOZ (Schultz et al., 2018). The general circulation model, ECHAM6.3, solves equations for surface pressure, vorticity, divergence and temperature (Stevens et al., 2013). ECHAM6.3 is further coupled with Hamburg Aerosol Model (HAM2.3), which is used to calculate all of the aerosol processes, including emissions, deposition, radiation and microphysics, within the global model (Tegen et al., 2019). HAM2.3 has also a comprehensive parameterization for both modal (M7, Tegen et al. (2019)) and sectional (SALSA, Kokkola, Kuhn, et al. (2018)) microphysics representations for the aerosol population and in this study the sectional approach was used.

### 2.2 MEGAN v2.1

**Figure 1:**
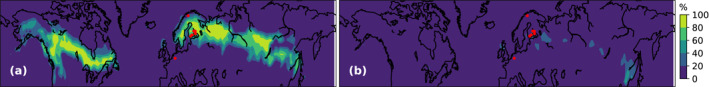
Needleleaf evergreen boreal (a) and broadleaf deciduous boreal (b) tree plant functional types from MEGAN. The percentage is a representation from the total land cover of the Earth. The red dots represent the locations of the field measurements, which were used to determine the average of healthy and stressed emission factors of these trees.

In order to simulate how changes in biogenic VOCs affect aerosol‐radiation and aerosol‐cloud‐interactions, Model of Emissions of Gases and Aerosols from Nature v2.1 (MEGAN) was used in ECHAM‐HAMMOZ simulations. MEGAN was used as it provides the plant functional types (PFTs) and emission factors (EFs) of several different VOCs to ECHAM‐HAMMOZ, which makes it easy to modify these PFTs and EFs for monoterpenes when simulating the plant damage effect on VOC emissions. MEGAN uses land cover, ambient meteorological properties, and atmospheric chemical composition as inputs estimating the net emissions of gases and aerosols from different types of terrestrial vegetation into the atmosphere (Guenther et al., 2012). In MEGAN there are 16 different PFTs which cover the whole land area of the Earth (Guenther et al., 2012; Wullschleger et al., 2014). These PFTs have different EFs for different chemical compounds which are released to the atmosphere as VOCs. In this study, we focus on changes in VOC emissions due to plant damages to needleleaf evergreen boreal (NEB) and broadleaf deciduous boreal (BDB) trees. The percentage of needleleaf evergreen boreal and broadleaf deciduous boreal trees from the total land cover of the Earth are presented in Figure 1. In addition, we will analyze the changes in atmospheric aerosol load as well as the resulting changes in aerosol‐cloud‐interactions.

We will study changes in monoterpene emissions, due to insect herbivory, as the EFs for these trees and compound were readily available from several measurement studies (Achotegui‐Castells et al., 2013; Blande et al., 2007, 2009, 2010; Brilli et al., 2009; Clavijo McCormick et al., 2014; Copolovici et al., 2011, 2017; Faiola & Taipale, 2020; Faiola et al., 2018; Ghimire et al., 2016; Ghirardo et al., 2012; Heijari et al., 2011; Joutsensaari et al., 2015; Kari et al., 2019; Kovalchuk et al., 2015; Litvak & Monson, 1998; Li et al., 2012; Maja et al., 2014; Mäntylä et al., 2008; Schaub et al., 2010; Yli‐Pirilä et al., 2016), and since monoterpenes are a major contributor to biogenic SOA formation over the boreal area (Hakola et al., 2006; Rinne et al., 2009). In addition, as the model includes only one sesquiterpene, the modification and analysis for sesquiterpenes and green leaf volatiles are left for future studies. EFs for monoterpenes averaged from several different measurement studies as well as the original MEGAN values for insect herbivory stressed and healthy needleleaf evergreen boreal and broadleaf deciduous boreal tree are presented in Table 1 (Achotegui‐Castells et al., 2013; Blande et al., 2007, 2009, 2010; Brilli et al., 2009; Clavijo McCormick et al., 2014; Copolovici et al., 2011, 2017; Faiola et al., 2018; Faiola & Taipale, 2020; Ghimire et al., 2016; Ghirardo et al., 2012; Heijari et al., 2011; Joutsensaari et al., 2015; Kari et al., 2019; Kovalchuk et al., 2015; Li et al., 2012; Litvak & Monson, 1998; Maja et al., 2014; Mäntylä et al., 2008; Schaub et al., 2010; Yli‐Pirilä et al., 2016). The averaged EFs were obtained from several field and laboratory experiments and the locations of the field measurements are marked as red dots in Figure 1.

**Table 1:** Emission Factors of Monoterpene for Healthy and Insect Herbivory Stressed Needleleaf Evergreen Boreal and Broadleaf Deciduous Boreal Trees Based on Measurements and the Original MEGAN Values

PFT	Healthy $\left(\frac{\mu g}{m^{2}h}\right)$	Stressed $\left(\frac{\mu g}{m^{2}h}\right)$	MEGAN $\left(\frac{\mu g}{m^{2}h}\right)$
Needleleaf evergreen boreal	4,500	40,811	1270
Broadleaf deciduous boreal	7,700	21,000	840

As the measurement based EFs of monoterpene for healthy and stressed plants are much higher than the original MEGAN values, we scaled the values to be in line with the MEGAN values. The values for the measured healthy plants EFs were scaled to match those of the original EFs in MEGAN. The measurement based EFs for stressed plants were scaled with the same scaling factor as the measurement based EFs for the healthy plants. Thus, the EFs of monoterpenes used in this study were 1,270 $\frac{\mu g}{m^{2}h}$ and 11,518 $\frac{\mu g}{m^{2}h}$ for the healthy and stressed needleleaf evergreen boreal trees and 840 $\frac{\mu g}{m^{2}h}$ and 2,291 $\frac{\mu g}{m^{2}h}$ for the healthy and stressed broadleaf deciduous boreal trees.

### 2.3 Simulations

In order to analyze the effects of insect herbivory caused plant stress VOC emissions on the atmosphere and radiative balance of the Earth, we conducted 6 different simulations using the ECHAM‐HAMMOZ global aerosol‐climate model. In the simulations, the fraction of stressed plants in the needleleaf evergreen boreal and broadleaf deciduous boreal trees were varied. The percentages were 0, 10, 25, 50, 75, and 100% stressed trees which represent different scenarios of biotic stress in plants caused by insect herbivory outbreaks. The simulation with 0% of plants stressed represents the base simulation using the default monoterpene EFs described in the model. The simulation with 10% of plants stressed (Stress10) represents the background aerosol (Kozlov & Zvereva, 2018). The simulation with 25% of plants stressed (Stress25) is a representation of the current day when the outbreaks of insect herbivore infestations are high (Michel et al., 2018). The simulation with 50% of plants stressed (Stress50) is a representation of a future scenario where current day infestation ourbreaks are increased (Venäläinen et al., 2020). The simulation with 75% of plants stressed (Stress75) was done to verify if there is a linear correlation between plant stress and SOA burden, clouds and radiative effects. The simulation with 100% of plants stressed (Stress100) is a representation of the upper bound of stress percentages. Table 2 summarizes the simulations used in this study with simulation names, their details and descriptions. All of the simulations were for a 10‐year period starting from January 2000 ending in December 2009. All simulations were preceded with a 1‐year spin‐up period. The data was simulated with 3‐hourly output throughout the period. From the 3‐hourly data, we calculated monthly means for the model values. The emissions were obtained from the CEDS (Community Emissions Data System) emission inventories and for all simulated years we used the monthly mean emissions from year 2010 (Hoesly et al., 2018). The sea surface temperature (SST) and sea ice cover (SIC) were prescribed and were obtained from monthly mean climatologies from AMIP (Atmospheric Model Intercomparison Project) (Taylor et al., 2012). In this study, we performed atmosphere‐only simulations with fixed SST, but freely evolving atmosphere. This means that the model accounts for aerosol‐radiation interactions (ARI), aerosol‐cloud interactions (ACI) and rapid adjustments, but not for climate feedbacks. The aerosol‐climate interactions are two‐way in the sense that possible climate (cloud) modifications also affect aerosol processes, most prominently aerosol removal and aerosol transport. Because land surface temperature is not fixed, VOC emissions may also change to some extent with changing aerosol loads. It should be noted that the simulations in this study are ideal and in reality the plant damages, due to herbivore infestation, are not assumed for the whole area simultaneously. However, the strongest effects are expected to be local and thus these simulations give indication where radiation and clouds are most susceptible to plant damage induced increase in SOA.

**Table 2:** Simulations Used in This Study

Simulation name	Details	Description
Stress0	0% stressed and 100% healthy NEB and BDB trees	Base simulation
Stress10	10% stressed and 90% healthy NEB and BDB trees	Background simulation
Stress25	25% stressed and 75% healthy NEB and BDB trees	Current day high outbreak simulation
Stress50	50% stressed and 50% healthy NEB and BDB trees	Increased current day high outbreak simulation
Stress75	75% stressed and 25% healthy NEB and BDB trees	Linearity check simulation
Stress100	100% stressed and 0% healthy NEB and BDB trees	Upper bound simulation

**Figure 2:**
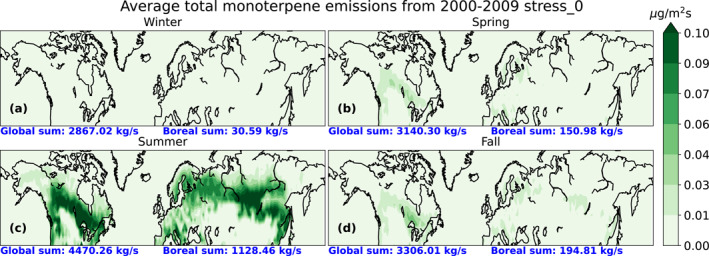
Seasonal average total monoterpene emission from years 2000 to 2009 simulated with the base simulation with the average global and boreal area sums for winter (December, January, and February) (a), spring (March, April, and May) (b), summer (June, July, and August) (c) and fall (September, October, and November) (d).

Figure 2 shows the seasonal averaged total monoterpene emissions from the base simulation as an average over 10‐year period as well as the boreal area and global sum for winter (December, January, and February), spring (March, April, and May), summer (June, July, and August) and fall (September, October, and November). In Figure 2 the total monoterpene emissions for both the whole globe and the boreal area are highest in summer (Figure 2c). In addition, the monoterpene emissions from the needleleaf evergreen boreal and broadleaf deciduous boreal trees are highest during summer. Thus in this study, we focused only on the summer season, as during this period, emissions due to biotic stress will have the greatest effect on aerosol forcing.

**Figure 3:**
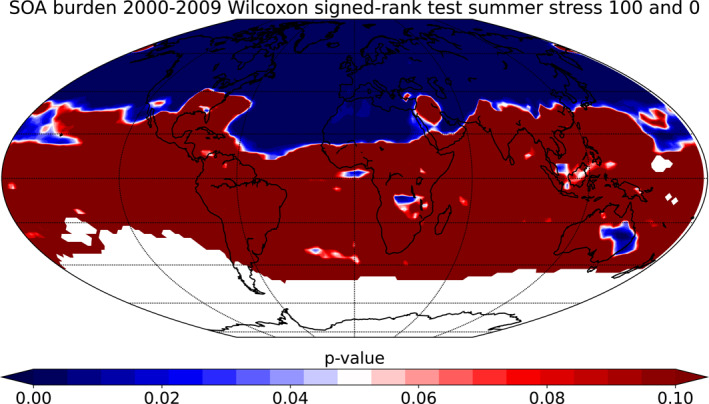
Wilcoxon signed‐rank test of secondary organic aerosol (SOA) burden from summer from 10‐year period between the upper bound and base simulations.

First, we limited the study area to a region where SOA burdens from plant emitted VOCs are significantly perturbed in the simulation with 100% plant stress. This was done using the Wilcoxon signed‐rank test which can be used to test if two related data sets come from a same distribution. The hypothesis of the test is that if the *p*‐value is high (more than 0.05) the two data sets do not have statistically significant difference. In addition, if the *p*‐value of the test is low (less than 0.05) there is a significant statistical difference between the two data sets (Wilcoxon, 1945). We used the Wilcoxon signed‐rank test to see what areas of the Earth would be most sensible to analyze in terms of changes in SOA burden between the base simulation and the simulation where 100 % of the plants are stressed. Wilcoxon signed‐rank test of SOA burden from summer months from 10‐year period between the upper bound and base simulation is presented in Figure 3. The large white areas are made to be unpresentable Not‐a‐Number values as they represent the areas where the SOA burden in both of the simulations is fairly small (lower than 2 × 10^−6^ kg/m^2^). In the Wilcoxon signed‐rank test between the upper bound and base simulation the most statistically significant difference (*p*‐value < 0.05) is achieved from 40° latitude northward. In addition, aerosol forcing is usually strongly dependent on the areas where aerosols are emitted and on the large ocean areas the dimethylsulfide and sea salt causes interference to the analysis. Thus, in our analysis we only focused on the Northern Hemisphere from 40° latitude northward and only on the land areas.

When studying cloud properties, it can be difficult to distinguish the actual signal from the noise caused by large temporal and spatial variability. In addition, as the length of the winter is long especially in the Northern Hemisphere the snow and ice covers can affect the surface albedo, even during summer months, causing a strong spatial and temporal fluctuation in aerosol radiative effects. Thus, in order to better separate the changes in cloud properties due to changes in VOC emissions, we conducted Gaussian filtering to smooth the 2D data in the results section following the approach of Miinalainen et al. (2021).

## 3 Results

We investigated the effect of elevated plant stress emissions on SOA formation, cloud properties, and radiative effects by analyzing simulation results for SOA burden, CDNC at cloud top, and clear‐sky and all‐sky shortwave RF at TOA. To evaluate regional variation of this effect, we plotted map figures from 40° latitude upward illustrating the absolute difference in SOA burden, CDNC at cloud top and shortwave RF between the base simulation and the plant stress simulations. In addition, to test the linearity of the increase or decrease in these variables, we generated a box plot from the field mean of the land area values for these variables.

First we investigated how increasing plant stress of the needleleaf evergreen boreal and broadleaf deciduous boreal trees affects the SOA formation, due to increasing VOC emissions. This was done by analyzing the difference in SOA burden between the base simulation and simulations where the monoterpene emissions of the trees were increased, over the inspected area.

**Figure 4:**
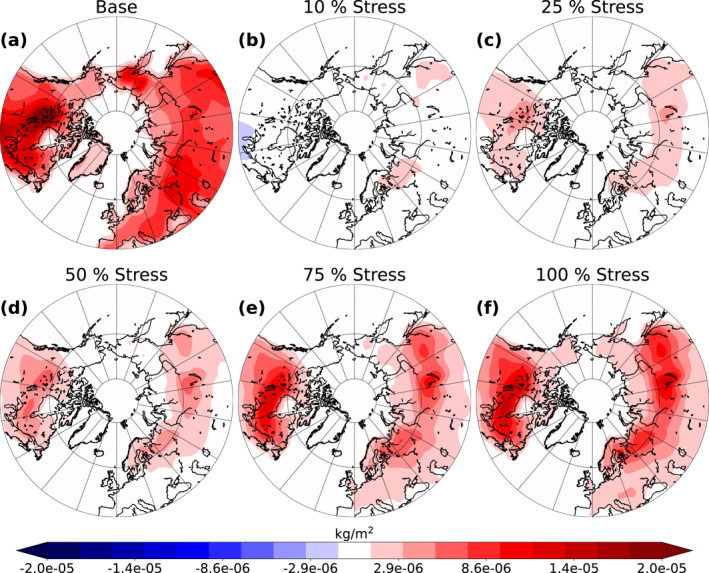
Absolute mean value of the base simulation (a) and absolute difference between the base simulation and the different stressed simulations (10 % (b), 25 % (c), 50 % (d), 75 % (e) and 100 % (f)) of mean secondary organic aerosol (SOA) burden, over land areas, from summer over 10‐year period.

Figure 4 shows the mean SOA burden in the base simulation and the absolute difference of the mean SOA burden, over land areas, between the stressed simulations and the base simulation for the summer period over 10 simulation years. There is an increase in the SOA burden when the stress imposed to needleleaf evergreen boreal and broadleaf deciduous boreal trees is increased. The increase is strongly dependent on the area where the majority of the aforementioned trees lay. The largest increase is in central Canada, Scandinavia, and throughout central Russia. There is no notable difference in the Stress10 simulation compared to the base simulation (Figure 4b), but already in Stress25 (Figure 4c) there is a significant increase in the SOA burden (approximately 5 × 10^−6^ kg/m^2^ at maximum which is approximately 50% higher than in the base simulation). The Stress100 simulation shows an increase of approximately 1.5 × 10^−5^ kg/m^2^ at maximum which is approximately 120% higher than in the base simulation (Figure 4f). In addition, the increase in SOA burden seems to be linear when going to larger stress values. This elevation in the SOA burden is due to higher monoterpene emissions from the trees of interest which in turn increases the SOA production via oxidation.

**Figure 5:**
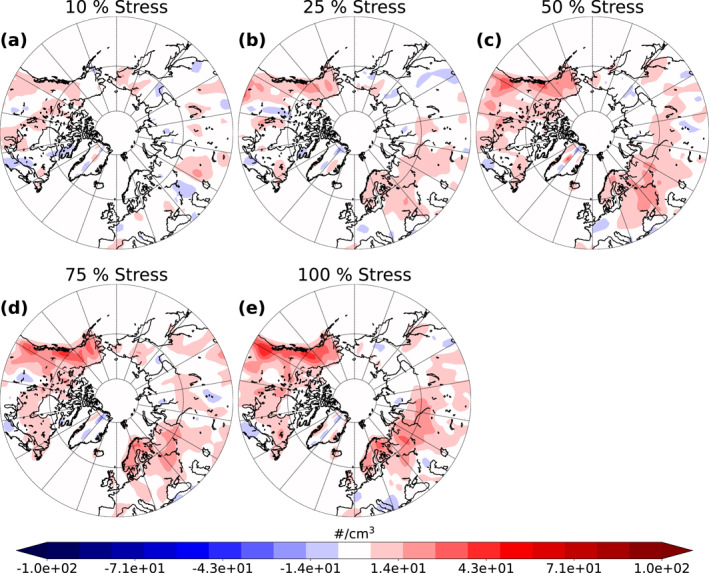
Absolute difference between the base simulation and the different stressed simulations (10 % (a), 25 % (b), 50 % (c), 75 % (d) and 100 % (e)) of mean land area cloud top cloud droplet number concentration (CDNC) weighted with cloud time from summer over 10‐year period.

As explained above, enhanced SOA formation increases the number concentration of particles acting as CCN thus modifying cloud properties (Kuang et al., 2009; Kulmala, Nieminen, et al., 2014; Yli‐Juuti et al., 2021). To see the effect of increase in SOA formation on cloud properties we studied the changes in CDNC at cloud top. Figure 5 presents the absolute difference in the mean cloud top CDNC, weighted with cloud time, over land areas between the stressed simulations and the base simulation for the summer period over 10 simulation years. There are only slight differences (approximately 20 #/cm^3^ increase at maximum which is approximately 30% higher than in the base simulation) in cloud top CDNC between the Stress10 simulation and the base simulation (Figure 5a). However, when the percentage of stressed trees increases, the differences become more distinguishable. Between the base simulation and the Stress100 simulation (Figure 5e) the increase is approximately 43 #/cm^3^ at maximum which corresponds to approximately 50% increase. Highest increase is seen in the western parts of Canada and Alaska as well as Scandinavia and central Russia. CDNC increases with increasing fraction of stressed trees and the increase in our simulations is quite linear. However, the change in CDNC does not change significantly when altering the stress percentage from 75 to 100 than from other stress percentages. This saturation in CDNC and the fact that the highest increases in CDNC do not correspond well with the places where we observed the highest increase in SOA, are due to CDNC susceptibility to CCN which decreases with increasing CCN concentrations (Carslaw et al., 2013).

**Figure 6:**
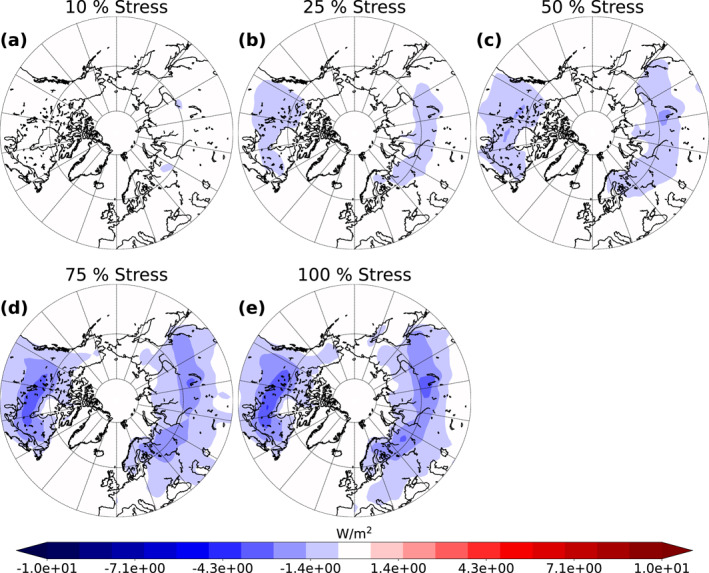
Mean land area clear‐sky top of the atmosphere (TOA) shortwave radiative forcing (RF) (aerosols) between the base simulation and the different stressed simulations (10% (a), 25% (b), 50% (c), 75% (d) and 100 % (e)) from June to August from 10‐year period.

**Figure 7:**
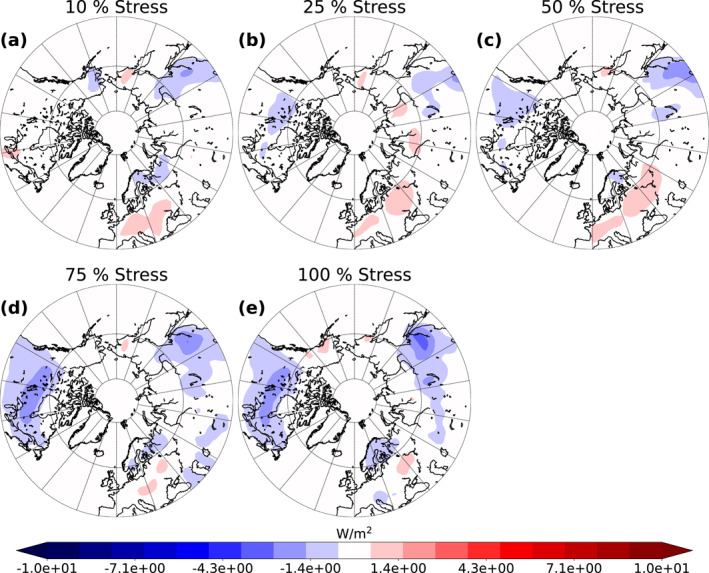
Mean land area all‐sky top of the atmosphere (TOA) shortwave radiative forcing (RF) (aerosols) between the base simulation and the different stressed simulations (10% (a), 25% (b), 50% (c), 75% (d) and 100 % (e)) from June to August from 10‐year period.

Our simulations also show that change in SOA has an effect on the RF via increased scattering of solar radiation by aerosols. To investigate the magnitude of this effect, we studied the clear‐sky and all‐sky shortwave RF due to aerosol‐radiation interactions. The mean clear‐sky and all‐sky shortwave RF due to increased VOC emissions over summer months for 10 simulation years are presented in Figures 6 and 7. The RF is calculated as *RF* = *RF*_ari,stress_ − *RF*_ari,base_, where *RF*_ari,stress_ is the RF due to aerosol‐radiation interactions at TOA from simulations where the plant stress was increased and *RF*_ari,base_ is the RF due to aerosol‐radiation interactions at TOA from the base simulation. The *RF*_ari_ is calculated as *RF*_ari_ = *F*_aerosols_ − *F*_no___aerosol_, where *F*_aerosols_ is the net radiative flux including aerosols and *F*_no___aerosols_ is the net radiative flux without aerosols (Ghan et al., 2012). These calculations do not include the aerosol‐cloud interactions and in this study, we do not address the RF due to aerosol‐cloud interactions as the actual signals were hard to detect from the variability caused by clouds even with the Gaussian filtering (Kühn et al., 2020).

**Figure 8:**
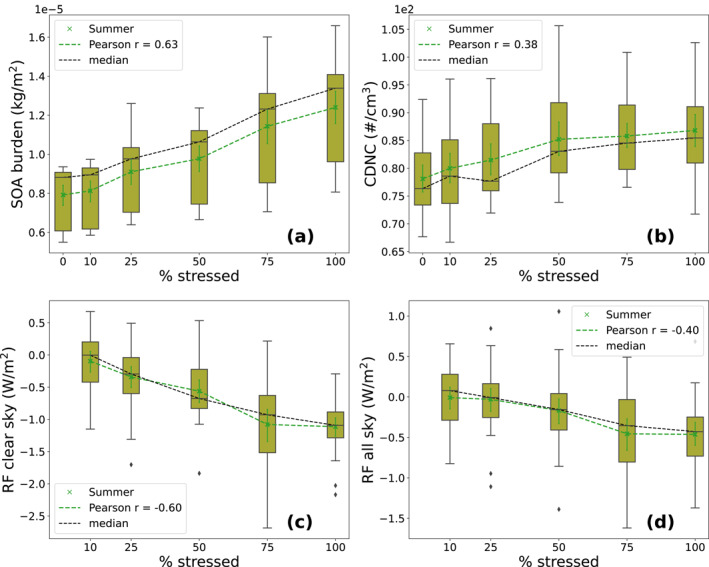
Box plots of field mean land area secondary organic aerosol (SOA) burden (a), cloud top cloud droplet number concentration (CDNC) weighted with cloud time (b) and clear‐sky (c) and all‐sky (d) top of the atmosphere (TOA) shortwave radiative forcing (RF) from 40 to 90° latitude region for summer from 10‐year period for all of the simulations. The boxes represent the interquartile range (IQR) which shows the middle of 50% of the values (lower end of the box represents the 25th percentile and upper end 75th percentile). Black whiskers outside the boxes represent the values outside the middle 50% and the tips represent the minimum and maximum values (excluding outliers). Black vertical lines and dashed lines represent the median values. Green crosses and dashed lines represent the mean values and the green whiskers around the mean values represent the error in mean calculations. The black diamonds represent the outliers in the data.

The negative clear‐sky RF (Figure 6) enhances with increasing stress percentage. This implies that there is more outgoing shortwave radiation in stressed simulations than there is in the base simulation. The effect is more obvious over areas where the majority of needleleaf evergreen boreal and broadleaf deciduous boreal forests are located. Thus, areas most affected by the negative RF are Canada, northern parts of US, Scandinavia, and Russia with the strongest negative forcing (approximately −4.3 W/m^2^) in the Stress100 simulation (Figure 6e). The decrease in RF seems to be quite linear when increasing the stress percentage. We can see similar linear decrease in all‐sky RF (Figure 7) as in clear‐sky RF (Figure 6), but the change in all‐sky RF is less pronounced. This is due to calculation of all‐sky values which take into account the values in the global model grid box where there are clouds, thus leaving less radiation for the aerosol particles to scatter. In addition, it causes more variation to the data. There are only slight differences (mainly due to noise) in the RF from 10, 25, and 50% stress simulations (Figures 7a–7c). However, the strongest negative all‐sky forcing is over Canada and northern parts of US which is approximately −2 W/m^2^ in Stress75 and Stress100 simulations (Figures 7d and 7e). In addition, there is a strong negative forcing (approximately −3 W/m^2^) in the Stress100 simulation (Figure 7e)) in eastern parts of Asia as well as slightly negative forcings over Scandinavia (approximately 1 W/m^2^). The linear dependence between the SOA burden, CDNC and the radiative effects can be more clearly seen from box plots of the 10‐year period monthly mean values as a field mean from the land area from 40 to 90° latitude region which is presented in Figure 8.

There is a moderate correlation with the land area SOA burden and the extent of stress. The mean and median of SOA burden experience only a minor increase (mean increase of approximately 0.1 × 10^−6^ kg/m^2^) when changing from 0 to 10% stress but all in all the increase is quite linear when going to higher stress percentages despite the fact that there is quite a strong deviation in the SOA burden which can be seen from the size of the boxes and black whiskers. Thus, increasing the plant stress enhances the VOC emissions and further the SOA production. The strongest increase in SOA burden (between base and Stress100 simulation) corresponds to approximately 95  % increase in field mean land area SOA mass concentration at surface which was approximately 1.9 μg/m^3^ in the base simulation and approximately 3.7 μg/m^3^ in the Stress100 simulation (not shown here). CDNC at cloud top increases with increasing stress percentage as well. However, the increase is not as linear as with SOA burden. There is only a weak correlation between plant stress and CDNC which can be mainly due to large variations in the data. However, there is an increase in mean values of CDNC especially at lower stress percentages (0%–50%). This is consistent with the increase in the SOA burden which increases the amount of particles acting as CCN. There is a moderate negative correlation between the extent of plant stress and TOA clear‐sky shortwave RF over land areas. This is well in line with the SOA burden as the SOA increases in the atmosphere it causes stronger negative RF due to aerosol‐radiation interactions. In addition, the TOA all‐sky shortwave RF over land areas becomes more negative with increasing plant stress, but the correlation is weaker than for the clear‐sky RF. This is because clouds mask part of aerosol forcing.

## 4 Conclusions

We investigated the effects of biotic stress, due to herbivore infestation, on needleleaf evergreen boreal and broadleaf deciduous boreal trees in terms of changes in SOA burden, CDNC, and aerosol radiative effects. We only included insect herbivory stress, in this study, as there are enough studies on that subject to make some initial estimates of stressed EFs. Pathogens are also very important, but there is very little quantitative information on the impact of pathogens on biogenic VOC emission rates. We used the ECHAM‐HAMMOZ global aerosol‐climate model with SALSA microphysics scheme and MEGAN v2.1 biogenic emission model to evaluate how the percentage of plant damage changes the simulated SOA burden, CDNC at cloud top and radiative effects compared to a simulation where plants were assumed to be healthy.

We found out that increasing levels of biotic plant stress elevated the SOA burden. The most considerable increase was over the areas where there were most of the needleleaf evergreen boreal and broadleaf deciduous boreal trees are located. In addition, the largest increase in SOA burden (up to 1.4 × 10^−5^ kg/m^2^) was between the base simulation and the simulation where 100% of the plants were stressed. The increase in SOA burden was due to larger emissions of monoterpenes of needleleaf evergreen boreal and broadleaf deciduous boreal trees which enhances the SOA formation. As the stress percentage increased there was also an increase in CDNC at cloud top. The main increase was at the areas where there was an elevation in SOA burden, but also at western parts of Canada and Alaska. The increase was at maximum (up to 40 #/cm^3^) between the base simulation and the simulation where 100  % of the plants were stressed. As SOA formation increases, due to increasing plant stress, it causes increment in the particles which can act as CCN and thus an elevation in CDNC at cloud top. This increase in CDNC can have a potential to change the cloud albedo which can lead to enhanced cooling effect of the clouds (Twomey, 1974).

The clear‐sky shortwave RF became strongly negative when the stress percentage was increased compared to the base simulation. In addition, the negative forcing is stronger over boreal forests. The simulation where 100% of the plants were stressed showed the strongest negative forcing (up to −4 W/m^2^) for the clear‐sky case. Negative all‐sky shortwave RF was also enchanced when increasing the fraction stressed plants. However, the effect was weaker than in clear‐sky case. The strongest negative forcing was in the simulation where 100% of the plants were stressed over eastern parts of China (up to −3 W/m^2^). The increase in the negative shortwave RF was due to increase in SOA formation, with increasing stress percentage, which affects the aerosol‐radiation interactions by reflecting more of the incoming solar radiation. These values are comparable to values given in the latest Assessment Report (AR6) of the Intergovernmental Panel on Climate Change (IPCC, 2021) where the multi‐model mean net effective RF due to aerosol changes between 1850 and recent‐past (1995–2014), in the boreal region, range between 0 and −6 W/m^2^. The strongest negative effect (between −4.5 and −6 W/m^2^) in the report is in central Russia which is mainly due to forest fires (IPCC, 2021).

Field mean SOA burden from 40 to 90° latitude over land areas showed a moderate correlation with increasing the fraction of stressed trees. Although there was a strong deviation in the SOA burden values, the mean and median values increase linearly with the stress percentage. This indicates that increasing monoterpene emissions of boreal trees increases SOA formation linearly. CDNC at cloud top, on the other hand, showed only a weak correlation with the stress percentage. The increase in mean CDNC at cloud top was linear when the stress percentages increased from 0 to 50  %, but after that the values saturated at approximately 85 #/cm^3^ because CDNC becomes less susceptible with increasing CCN (Carslaw et al., 2013). The moderate negative correlation between clear‐sky shortwave RF and increasing plant stress percentage is consistent with the increase in SOA burden. As the stress percentage increased, the SOA formation increased linearly which further induced stronger negative RF as there is a shift in aerosol particle size distribution which enhances the reflection and scattering of the incoming solar radiation. However, the all‐sky shortwave RF had only weak negative correlation with increasing plant stress percentage. This is due to clouds which also reflect the incoming solar radiation masking the aerosol radiative effect.

As stated in Section 2, the simulations where 25% and 50 % of the plants are stressed represent the current day high insect outbreak and future increased high insect outbreak scenarios, respectively (Michel et al., 2018; Venäläinen et al., 2020). On average the field mean SOA burden increases approximately 7.4% causing an increase of approximately 4.6% in CDNC between the current day and future high insect outbreak scenarios. In addition, on average the clear‐sky RF shows approximately −0.22 W/m^2^ and all‐sky RF −0.14 W/m^2^ stronger negative effect between these scenarios. In SMEAR II station at Hyytiälä, Finland, the estimated growth in negative effect of RF due to aerosol‐radiation interactions to be −1.15 W/m^2^ for clear‐sky and −0.33 W/m^2^ for all‐sky case for every degree of celsius increase over boreal forests (Yli‐Juuti et al., 2021). Thus, in our simulations, the RF change from current day to future scenario corresponds to values of approximately 0.2° and approximately 0.4° increase in temperature for the clear‐sky and all‐sky cases, respectively. Using these stress percentages, the increase in SOA concentrations and their effect to CDNC and RF appears to be almost linear. It can be assumed that other factors increasing SOA through increasing VOC emissions (e.g., temperature driven increase VOC emissions), would be additive to these biotic stresses caused by herbivore infestation. This effect would also apply for other abiotic factors such as increased drought episodes or salinity. Although in this study, we focus on monoterpenes, biotic stresses affect the composition of the emitted VOC through changes in isoprene and sesquiterpene emissions which in turn modifies the hygroscopicity and CCN activity of the formed SOA (Zhao et al., 2017). However, isoprene emissions do not dominate as strongly as monoterpenes, in the boreal region, which was the area of focus in this study (Hantson et al., 2017). In addition, there are no existing sesquiterpene volatility basis set (VBS) parameters that adequately predict SOA formation from sesquiterpene oxidation (Barsanti et al., 2013). Nevertheless, including biotic stress effects on VOC composition and to isoprene and sesquiterpene emissions, would extend our understanding of the climate effects of plant stress induced aerosol and should be a topic for future studies.

To conclude, increasing monoterpene EFs for needleleaf evergreen boreal and broadleaf deciduous boreal trees (which simulates the plant stress caused by biotic stress factors) increases SOA formation through oxidation processes in ECHAM‐HAMMOZ global aerosol‐climate model. The increase in SOA burden is moderately dependent on increase in stress percentage of the plants. The increase in SOA burden affects the cloud formation by increasing the amount of particles acting as CCN and thus increasing CDNC at cloud top. In addition, the increase in SOA burden decreases the shortwave RF, which is due to increase in aerosol particles which reflect the incoming solar radiation. Even though our simulations are ideal and in reality the plant damages are not assumed for the whole area simultaneously, the global climate model development should include the effects of biotic plant stress in VOC emissions, to some extent, as this could have a significant effect on SOA formation, clouds and radiative effects.

## Data Availability

The data and codes for reproducing the figures and the settings for the simulations are openly available (E. Holopainen et al., 2022). All other input files are ECHAM6‐HAMMOZ standard and are available from the HAMMOZ repository (HAMMOZ consortium, 2020). The stand‐alone zero‐dimensional version of SALSA2.0 is distributed under the Apache‐2.0 licence at http://www.apache.org/licenses/LICENSE-2.0 and the code is openly available (Kokkola, Tonttila, et al., 2018). The model data can be reproduced using the model revision r6431 (HAMMOZ consortium, 2020). The ECHAM6‐HAMMOZ model is made available to the scientific community under the HAMMOZ Software Licence Agreement, which defines the conditions under which the model can be used. The licence can be downloaded from https://redmine.hammoz.ethz.ch/attachments/291/License_ECHAM-HAMMOZ_June2012.pdf.
